# Physical healthcare gap among patients with severe mental illness through the COVID-19 pandemic. Preliminary results from a real-world investigation in Lombardy, Italy

**DOI:** 10.1192/j.eurpsy.2024.216

**Published:** 2024-08-27

**Authors:** C. Conflitti, M. Monzio Compagnoni, G. Corrao, A. Lora

**Affiliations:** ^1^Statistics and Quantitative Methods, University of Milan-Bicocca; ^2^National Centre of Healthcare Research & Pharmacoepidemiology, Milan; ^3^Department of Mental Health, ASST Lecco, Lecco; ^4^University of Milan-Bicocca , Milan, Italy

## Abstract

**Introduction:**

Patients suffering from mental disorders tend to be less adherent to the recommended therapies. Moreover, the COVID-19 pandemic had a global impact on physical and social well-being, which turned out stronger in the most fragile patients, like those with a mental condition.

**Objectives:**

To assess whether the COVID-19 pandemic influenced the physical healthcare gap between patients with and without severe mental illness (SMI) treated for chronic conditions.

**Methods:**

Data were retrieved from Healthcare Utilization Databases of Lombardy region (Italy). Prevalent users of antihypertensive drugs, statins or antidiabetic drugs, receiving healthcare in Lombardy during 2020, were identified. Among them, those with a previous diagnosis of schizophrenic or bipolar disorder were selected and matched with up to 3 patients without any sign of mental disorder by sex, age and number of contacts with the NHS during the previous year. 3 cohorts (not necessarily independent) were formed.

High adherence to specific recommended drug therapies and discontinuation during 2020 were evaluated.

Association between presence of SMI and high adherence was evaluated by using a log-binomial model (risk ratios, RR with 95% CI); a Cox model (hazard ratios, HR) was used for discontinuation.

As comparison, same analyses were performed to the cohorts of prevalent users in 2019, to evaluate the impact of the COVID-19 pandemic. Results were stratified according to the type of mental disorder.

**Results:**

36'436, 14'136 and 12'597 prevalent users of antihypertensives, statins or antidiabetics respectively were identified, of which 25% with SMI (9'109, 3'536 and 3'152 respectively).

During the pandemic period, for all the three cohorts, patients with mental illness had 10% lower probability of being adherent to the recommended drug therapies.

The association between SMI and discontinuation was significant and varied among the three cohorts, with HR (95% CI): 1.27 (1.21; 1.33) for antihypertensives users, 1.16 (1.07; 1.26) for antidiabetics users and 1.08 (1.01; 1.16) for statins users.

Compared with 2019 the gap remained similar, except for discontinuation of antidiabetics, where the gap diminished from 34% in 2019 to 16% in 2020.

No differences between the two mental disorders were found.

**Conclusions:**

Results show that suffering from a mental disorder in people with chronic physical conditions affects their adherence to recommended drug therapies. During the pandemic period, the restrictive measures adopted may have led to a better care by family members, counteracting any increase in the gap.

The healthcare gap in patients suffering from mental illness remains an unsolved problem of primary importance for public health.

**Disclosure of Interest:**

None Declared

